# On the rotational equations of motion in rigid body dynamics when using Euler parameters

**DOI:** 10.1007/s11071-015-1995-3

**Published:** 2015-02-28

**Authors:** Karim Sherif, Karin Nachbagauer, Wolfgang Steiner

**Affiliations:** 1 Johannes Kepler University of Linz, Altenbergerstr. 69, 4040 Linz, Austria; 2 Faculty of Engineering and Environmental Sciences, University of Applied Sciences Upper Austria, Stelzhamerstr. 23, 4600 Wels, Austria

**Keywords:** Euler parameter, Rotational equations of motion, Generalized force vector, Gyroscopic moment

## Abstract

Many models of three-dimensional rigid body dynamics employ Euler parameters as rotational coordinates. Since the four Euler parameters are not independent, one has to consider the quaternion constraint in the equations of motion. This is usually done by the Lagrange multiplier technique. In the present paper, various forms of the rotational equations of motion will be derived, and it will be shown that they can be transformed into each other. Special attention is hereby given to the value of the Lagrange multiplier and the complexity of terms representing the inertia forces. Particular attention is also paid to the rotational generalized external force vector, which is not unique when using Euler parameters as rotational coordinates.

## Introduction

Multibody systems allow the modeling of entire systems of rigid and flexible bodies connected by joints and driven by forces and actuators. The most common and widely used type of rotational coordinates in multibody systems that treat the orientation of either rigid or flexible bodies are Euler angles. An alternative non-minimal representation of the orientation of a body is given by a so-called quaternionic parametrization. In recent years, quaternionic parametrizations found new attraction because they substantially simplify the mathematical formulation and they lead, in contrast to parametrization groups using three variables, to a singularity-free description of the rotation.

The present paper deals with the different forms of the rotational equations of motion of an unconstrained rigid body in multibody systems using Euler parameters. The latter parameters are unit quaternions, that is, a collection of four real parameters, which are not independent. In terms of Euler’s theorem [1], three of the four Euler parameters represent a rotation axis and the fourth is considered as single rotation about the latter axis. Since the four Euler parameters are not independent, one has to consider the quaternion constraint in the equations of motion. This is usually done by using the Lagrange multiplier technique [2] where the derivative of the unity of the Euler parameters is related to the angular velocity. A numerical time integration of the latter equations, however, would lead to errors in the unity of the quaternions. Therefore, it is essential to explicitly extend the equations by the unity of the quaternions yielding a set of DAEs instead of ODEs. In this case, the Lagrange multiplier has no physical meaning. Alternatively, one can find several approaches avoiding the Lagrange multiplier technique, e.g., in the work by Möller and Glocker [3], in which unconstrained quaternions are used. An alternative derivation of the quaternion equations of motion for a rigid body has been presented by Udwadia and Schutte [4]. In [5], the equations of motion have been derived firstly in terms of quaternions using Lagrange’s formalism in order to analyze constrained mechanical systems including non-redundant holonomic constraints. Moreover, the latter paper shows as well how the physical torque vector can be expressed as the generalized quaternion torque vector. Alternatively, in [6], a different form for the generalized quaternion torque vector is presented, and by analytically solving the equations of motion, it has been demonstrated that the Lagrange multiplier according to the unity of the quaternion constraint is equal to twice the rotational kinetic energy, see also [7].

Similar to the work in [6], Vadali [8] has demonstrated that Lagrangian equations can be properly reformulated, such that the value of the Lagrange multiplier is zero. Although the Lagrange multiplier is zero within the latter formulation, it would lead to an error in the numerical time integration if the unity of the Euler parameters is no longer explicitly considered in the equations as a result of the drift phenomenon. Therefore, one has to re-normalize the Euler parameters after each numerical time integration step on position level in order to fulfill the unity of the Euler parameters, see e.g., [3, 7, 9].

Although one can find different forms of the generalized quaternion torque vector and as well suggestions on the amplitude of the Lagrange multiplier when using Euler parametrization, the connection between the generalized quaternion torque vector and the Lagrange multiplier has not yet been presented in the open literature. In the present paper, particular attention is paid on various different forms of the generalized quaternion torque vector and their influence on the amplitude of the Lagrange multiplier in rotational dynamics.

Furthermore, in the present paper, the generalized force vector is split into two parts, one part takes into account the rotational dynamics of the body, while the second part does not influence the rotation of the body at all. This fact has been mentioned as well in the work by Udwadia and Schutte [4], in which the connection between the physically applied torque and the generalized torque associated to the Euler parameters has been derived. To be more precise, the derivation in [4] reveals that the generalized torque vector includes a component in the direction of the Euler parameter vector which does not contribute to the rotational dynamics of a rigid body.

The present paper shows that all the forms of the equations of motion presented herein lead to the same rotational dynamics of the body. The difference between the presented forms is given by the amplitude of the Lagrange multiplier and the mathematical complexity in the equations of motion. The Euler’s equations are the starting point of consideration in order to show that the Lagrange parameter concerning the Euler parameter constraint gets zero, contrary to the derivation in [8], in which the Lagrangian equations are reformulated leading to the Euler’s equations. Moreover, the derivation of the equations of motion for a single rigid body from d’Alembert’s variational principle in the present paper reveals various forms when using Euler parameters for the description of the rotation. Of particular importance is the presented derivation of the generalized quaternion torque vector in terms of the physically applied force vector, which can also be related to the approach presented in [6, 10]. In addition to the work in the latter mentioned publications, in the present paper the different forms of the generalized quaternion torque vector are analyzed leading to new insights and their influence on the Lagrange parameter will be emphasized herein.

## Kinematic description

This section defines the kinematic variables of interest, and recalls a number of fundamental kinematic relations, which are needed in the present paper. Fundamental derivations concerning Euler parameters and a collection of many useful quaternion identities are presented, see also [6].

In order to describe the position vector $\mathbf {r}^{p}$ of an arbitrary point $P$ on a rigid body in the floating frame of reference formulation [11] two different types of coordinate systems.

One is a time- and space-fixed inertial coordinate system and the second one is attached to the rigid body and, therefore, translates and rotates with the body. Thus, the absolute position vector of a point $P$ can be written as [11–13]

1 $$\begin{aligned} \mathbf {r}^{p}=\mathbf {R}+\mathbf {A}{\overline{\mathbf {u}}}^{p} \end{aligned}$$

where $\mathbf {R}$ is the position vector of the origin of the space-fixed coordinate system to the origin of the body coordinate system; the orthogonal rotation matrix $\mathbf {A}$ transforms quantities expressed in the body coordinate system into their counterparts in the space-fixed frame, and ${\overline{\mathbf {u}}}^{p}$ is the position vector of the point defined in the body coordinate system. Unless otherwise stated, throughout the present paper, quantities with an overbar are expressed in the body reference system. Note that any vector can, at any instant of time, be decomposed in either the space-fixed or the body coordinate system.

While the three translational coordinates of the vector $\mathbf {R}$ are needed to fix the location of a body, in the present paper a four-component vector ${\mathbf {p}}$ containing Euler parameters

2 $$\begin{aligned} {\mathbf {p}}^{T} = \left[ e_{0} \,\, \mathbf {e}^{T}\right] , \qquad \mathbf {e}^{T}= \left[ e_{1} \,\, e_{2} \,\, e_{3}\right] \end{aligned}$$

is used for the parametrization of the orientation of the body. The four quantities $e_{0},\, e_{1},\, e_{2}$ and $e_{3}$ represent the Euler parameters and are not independent, since they fulfill the following relation

3 $$\begin{aligned} e_{0}^{2}+\mathbf {e}^{T}\mathbf {e}=1 \end{aligned}$$

i.e.,

4 $$\begin{aligned} {\mathbf {p}}^{T}{\mathbf {p}}-1=0 \end{aligned}$$

In terms of the latter quaternions, the orthogonal rotation matrix $\mathbf {A}$, which transforms quantities from the body coordinate system into the global coordinate system, is given by [14]

5 $$\begin{aligned} \mathbf {A}=\left( 2e_{0}^{2}-1\right) \mathbf {I}+2\left( \mathbf {e}\mathbf {e}^{T}+e_{0}\widetilde{\mathbf{e}}\right) \end{aligned}$$

where $\mathbf {I}$ is an order-three identity matrix, and the skew-symmetric matrix $\widetilde{\mathbf{e}}$, with the axial vector $\mathbf {e}$, satisfies

6 $$\begin{aligned} \widetilde{\mathbf{e}}\mathbf {v}=\mathbf {e}\times \mathbf {v} \end{aligned}$$

for an arbitrary vector $\mathbf {v}$. The latter rotation matrix can also be written as [14]

7 $$\begin{aligned} \mathbf {A}={\mathbf {G}}{\mathbf {L}}^{T} \end{aligned}$$

with the pair of $3\times 4$ matrices ${\mathbf {G}}$ and ${\mathbf {L}}$ given by [14]

8 $$\begin{aligned} {\mathbf {G}}=\left[ \begin{array}{c@{}c@{}c@{}c} -e_{1} &{} e_{0} &{} -e_{3} &{} e_{2}\\ -e_{2} &{} e_{3} &{} e_{0} &{} -e_{1}\\ -e_{3} &{} -e_{2} &{} e_{1} &{} e_{0} \end{array}\right] =\left[ \begin{array}{c@{}c} -\mathbf {e},&\widetilde{\mathbf{e}}+e_{0}\mathbf {I}\end{array}\right] \end{aligned}$$

and

9 $$\begin{aligned} {\mathbf {L}}=\left[ \begin{array}{c@{}c@{}c@{}c} -e_{1} &{} e_{0} &{} e_{3} &{} -e_{2}\\ -e_{2} &{} -e_{3} &{} e_{0} &{} e_{1}\\ -e_{3} &{} e_{2} &{} -e_{1} &{} e_{0} \end{array}\right] = \left[ \begin{array}{c@{}c} -\mathbf {e},&-\widetilde{\mathbf{e}}+e_{0}\mathbf {I}\end{array}\right] \end{aligned}$$

A direct calculation reveals that

10 $$\begin{aligned} {\mathbf {G}}{\mathbf {G}}^{T}={\mathbf {L}}{\mathbf {L}}^{T}=\mathbf {I} \end{aligned}$$

while

11 $$\begin{aligned} {\mathbf {G}}^{T}{\mathbf {G}}={\mathbf {L}}^{T}{\mathbf {L}}=\mathbf {I}_{4}-{\mathbf {p}}{\mathbf {p}}^{T} \end{aligned}$$

where $\mathbf {I}_{4}$ denotes the $4\times 4$ identity matrix. In addition, ${\mathbf {G}},\, {\mathbf {L}}$ and ${\mathbf {p}}$ satisfy the following identities

12 $$\begin{aligned} {\mathbf {G}}{\mathbf {p}}=\mathbf {0},\qquad {\mathbf {L}}{\mathbf {p}}=\mathbf {0},\qquad \dot{\mathbf{G}}\dot{\mathbf{p}}=\mathbf {0},\qquad {\dot{\mathbf{L}}}\dot{\mathbf{p}}=\mathbf {0} \end{aligned}$$

Due to the orthogonality of the rotation matrix $\mathbf {A}$, we have

13 $$\begin{aligned} \mathbf {A}{}^{T}\mathbf {A}=\mathbf {I} \end{aligned}$$

The differentiation of the previous equation verifies that the matrix $\mathbf {A}{}^{T}{\dot{\mathbf{A}}}$ is skew-symmetric, since

14 $$\begin{aligned} \frac{\hbox {d}}{\hbox {d}t}\left( \mathbf {A}{}^{T}\mathbf {A}\right) =\mathbf {A}{}^{T}{\dot{\mathbf{A}}}+\dot{\mathbf{A}}{}^{T}\mathbf {A}=\mathbf {A}{}^{T}\dot{\mathbf{A}}+\left( \mathbf {A}{}^{T}\dot{\mathbf{A}}\right) ^{T}=\mathbf {0} \end{aligned}$$

Let us, therefore, define

15 $$\begin{aligned} {\widetilde{\overline{\varvec{\omega }}}}=\mathbf {A}{}^{T}\dot{\mathbf{A}} \end{aligned}$$

where the axial vector of ${\widetilde{\overline{\varvec{\omega }}}}$ may be interpreted as the angular velocity vector expressed in the body coordinate system. The skew-symmetric matrix ${\widetilde{\overline{\varvec{\omega }}}}$ can be alternatively written in terms of the matrix ${\mathbf {L}}$ and its first time derivative in the form

16 $$\begin{aligned} {\widetilde{\overline{\varvec{\omega }}}}=2{\mathbf {L}}{\dot{\mathbf{L}}}{}^{T} \end{aligned}$$

The kinematic equation that relates the time derivatives of the Euler parameters to the components of the angular velocity vector $\overline{\varvec{\omega }}$ can be written in the form [15]

17 $$\begin{aligned} {\overline{\varvec{\omega }}}=2{\mathbf {L}}\dot{\mathbf{p}} \end{aligned}$$

## Equations of motion

In this section, we recapitulate the derivation of the equations of motion for a single rigid body from d’Alembert’s variational principle and show that we end up with various forms of these equations if Euler parameters are used for the description of the rotation.

Assuming that the origin of the body coordinate system is attached to the center of mass of the body, d’Alembert’s principle for an unconstrained rigid body states that [16]

18 $$\begin{aligned} \delta \mathbf {R}^T\left( m{\ddot{\mathbf{R}}}-{\mathbf {f}}\right) + \delta {\overline{\varvec{\theta }}}^{T}\left( {\mathbf {J}}\dot{\overline{\varvec{\omega }}}+{\widetilde{\overline{\varvec{\omega }}}}{\mathbf {J}}{\overline{\varvec{\omega }}}-\overline{\mathbf {n}}-\overline{\mathbf {m}}\right) = 0 \end{aligned}$$

where $m$ denotes the mass of the rigid body and ${\mathbf {J}}$ its tensor of inertia, referred to the body coordinate system. The sum of all forces acting on the body is denoted by ${\mathbf {f}}$ and the sum of the moments induced by ${\mathbf {f}}$ is defined by $\overline{\mathbf {n}}$. Any other pure moments acting on the rigid body are defined by the vector $\overline{\mathbf {m}}$. Without loss of generality, we assume, from now on, that no pure moments act on the body.

Equation (18) must hold for any virtual displacement vector $\delta \mathbf {R}$ and for any rotation vector $\delta {\overline{\varvec{\theta }}}$, which may be expressed by Euler parameters in a similar way as ${\overline{\varvec{\omega }}}$ in Eq. (17):

19 $$\begin{aligned} \delta {\overline{\varvec{\theta }}}=2{\mathbf {L}}\delta {\mathbf {p}} \end{aligned}$$

Hence, by substituting Eqs. (16), (17) and (19) into d’Alembert’s principle and considering only the rotational part (i.e., we set $\delta \mathbf {R}=0$), we obtain

20 $$\begin{aligned} \delta {\mathbf {p}}^T\left( 4{\mathbf {L}}^{T}{\mathbf {J}}{\mathbf {L}}\ddot{\mathbf{p}}+8{\mathbf {L}}^{T}{\mathbf {L}}{\dot{\mathbf{L}}}{}^{T}{\mathbf {J}}{\mathbf {L}}\dot{\mathbf{p}}-2{\mathbf {L}}^{T}\overline{\mathbf {n}}\right) = 0 \end{aligned}$$

However, due to the fact that Euler parameters are not independent quantities, we may not equate the expression in brackets to zero. Because of Eq. (4), the variation $\delta {\mathbf {p}}$ is constrained by the relation

21 $$\begin{aligned} \delta {\mathbf {p}}^T {\mathbf {p}} = 0 \end{aligned}$$

Hence, the zero term $\delta {\mathbf {p}}^T {\mathbf {p}}\lambda$ may be added to Eq. (20), yielding

22 $$\begin{aligned} \delta {\mathbf {p}}^T\left( 4{\mathbf {L}}^{T}{\mathbf {J}}{\mathbf {L}}\ddot{\mathbf{p}}+8{\mathbf {L}}^{T}{\mathbf {L}}{\dot{\mathbf{L}}}{}^{T}{\mathbf {J}}{\mathbf {L}}\dot{\mathbf{p}}-2{\mathbf {L}}^{T}\overline{\mathbf {n}}+{\mathbf {p}}\lambda \right) = 0 \end{aligned}$$

The Lagrange multiplier $\lambda$ may now be defined by the condition that the expression in brackets vanishes. This results in the following four equations for the four Euler parameters and the Lagrange multiplier $\lambda$:

23 $$\begin{aligned} 4{\mathbf {L}}^{T}{\mathbf {J}}{\mathbf {L}}\ddot{\mathbf{p}}+8{\mathbf {L}}^{T}{\mathbf {L}}{\dot{\mathbf{L}}}{}^{T}{\mathbf {J}}{\mathbf {L}}\dot{\mathbf{p}} +{\mathbf {p}}\lambda = 2{\mathbf {L}}^{T}\overline{\mathbf {n}} \end{aligned}$$

The missing fifth equation is the constraint equation, see Eq. (4). The term ${\mathbf {p}}\lambda$ represents the constraint force, whereby ${\mathbf {p}}$ is the transpose of the constraint Jacobian of the kinematic constraint ${\mathbf {p}}^{T}{\mathbf {p}} = 1$. It can be easily demonstrated that the Lagrange multiplier $\lambda$ in Eq. (23) is zero, see also [8]. For that purpose, we pre-multiply Eq. (23) with ${\mathbf {p}}^T$ and make use of ${\mathbf {L}}{\mathbf {p}}=\mathbf {0}$. This results in

24 $$\begin{aligned} {\mathbf {p}}^T{\mathbf {p}} \lambda = 0 \end{aligned}$$

which can only be true if $\lambda =0$, since ${\mathbf {p}}^T{\mathbf {p}}=1$. At this point, it should be mentioned that Eq. (23) could be obtained as well by inserting Euler parameter identities of Eqs. (16) and (17) into the well-known Newton Euler equations and adding the constraint force ${\mathbf {p}}\lambda$.

Another form of the equations of motion is obtained by eliminating the Lagrange multiplier from Eq. (23) by transforming the equation back to the three-dimensional space. This is achieved by pre-multiplying Eq. (23) with the $3\times 4$ matrix ${\mathbf {L}}$. Since ${\mathbf {L}}{\mathbf {p}}=\mathbf {0}$ and by making use of ${\mathbf {L}}{\mathbf {L}}^T=\mathbf {I}$, we get

25 $$\begin{aligned} 4{\mathbf {J}}{\mathbf {L}}\ddot{\mathbf{p}}+8{\mathbf {L}}{\dot{\mathbf{L}}}{}^{T}{\mathbf {J}}{\mathbf {L}}\dot{\mathbf{p}} = 2\overline{\mathbf {n}} \end{aligned}$$

Because we have now reduced the number of equations from four to three, the constraint equation, i.e., Eq. (4), must still be considered to determine the four Euler parameters. It should be noticed that Eq. (25) can also be derived from the original form of d’Alembert’s principle. Since Eq. (18) must hold for any virtual rotation $\delta {\overline{\varvec{\theta }}}$, we obtain the Newton Euler equations

26 $$\begin{aligned} {\mathbf {J}}{\dot{\overline{\varvec{\omega }}}}+{\widetilde{\overline{\varvec{\omega }}}}{\mathbf {J}}{\overline{\varvec{\omega }}}-\overline{\mathbf {n}}=\mathbf {0} \end{aligned}$$

and by substituting Eqs. (16, 17) for ${\overline{\varvec{\omega }}}$ and ${\widetilde{\overline{\varvec{\omega }}}}$, we end up with Eq. (25) again.

One can obtain a third form of the equations of motion by starting again with d’Alembert’s principle of Eq. (20). The latter equation is constrained by the relation $\delta {\mathbf {p}}^T {\mathbf {p}} = 0$. Therefore, we substitute the identity of Eq. (11), i.e., ${\mathbf {L}}^{T}{\mathbf {L}}=\mathbf {I}_{4}-{\mathbf {p}}{\mathbf {p}}^{T}$, in the second term on the left side of Eq. (20), which actually represents the gyroscopic moment. This results in

27 $$\begin{aligned}&\delta {\mathbf {p}}^T\big (4{\mathbf {L}}^{T}{\mathbf {J}}{\mathbf {L}}\ddot{\mathbf{p}}+8{\dot{\mathbf{L}}}{}^{T}{\mathbf {J}}{\mathbf {L}}\dot{\mathbf{p}}\nonumber \\&\quad -\,8{\mathbf {p}}{\mathbf {p}}^{T}{\dot{\mathbf{L}}}{}^{T}{\mathbf {J}}{\mathbf {L}}\dot{\mathbf{p}}-2{\mathbf {L}}^{T}\overline{\mathbf {n}}\big ) = 0 \end{aligned}$$

Since $\delta {\mathbf {p}}^T {\mathbf {p}} = 0$, the gyroscopic moment can be simplified to $8{\dot{\mathbf{L}}}{}^{T}{\mathbf {J}}{\mathbf {L}}\dot{\mathbf{p}}$ and, thus, the latter equation can be rewritten in a simpler form as

28 $$\begin{aligned} \delta {\mathbf {p}}^T\left( 4{\mathbf {L}}^{T}{\mathbf {J}}{\mathbf {L}}\ddot{\mathbf{p}}+8{\dot{\mathbf{L}}}{}^{T}{\mathbf {J}}{\mathbf {L}}\dot{\mathbf{p}}-2{\mathbf {L}}^{T}\overline{\mathbf {n}}\right) = 0 \end{aligned}$$

As it is the case in Eq. (20), one may not equate the expression in brackets to zero because the Euler parameters are not independent quantities. First one has to add the zero term $\delta {\mathbf {p}}^T {\mathbf {p}}\lambda$ to Eq. (28) and then the expression in brackets can be set to zero which leads to the following form of the equations of motion:

29 $$\begin{aligned} 4{\mathbf {L}}^{T}{\mathbf {J}}{\mathbf {L}}\ddot{\mathbf{p}}+8{\dot{\mathbf{L}}}{}^{T}{\mathbf {J}}{\mathbf {L}}\dot{\mathbf{p}}+{\mathbf {p}}\lambda = 2{\mathbf {L}}^{T}\overline{\mathbf {n}} \end{aligned}$$

which has been presented as well in [6]. The latter equation is constrained by ${\mathbf {p}}^{T}{\mathbf {p}}=1$, which always has to be satisfied. However, the latter form is simpler than Eq. (23), since it contains only third-order terms in the Euler parameters, whereas we have to deal with fifth-order expressions in Eq. (23). This is mainly based on the simplification of the gyroscopic moment. The value of the Lagrange multiplier of Eq. (29) can be computed by pre-multiplying Eq. (29) with ${\mathbf {p}}^T$ and making use of ${\mathbf {L}}{\mathbf {p}}=\mathbf {0}$ and ${\mathbf {p}}^T{\mathbf {p}}=1$. This results in

30 $$\begin{aligned} \lambda = -8{\mathbf {p}}^T{\dot{\mathbf{L}}}{}^{T}{\mathbf {J}}{\mathbf {L}}\dot{\mathbf{p}} \end{aligned}$$

Now, the Lagrange multiplier is no longer zero, and the constraint force accounts for the simplification made in the gyroscopic moment. Clearly, if Eq. (29) is transformed back to the three-dimensional space by simply pre-multiplying the latter equation with the $3\times 4$ projection matrix ${\mathbf {L}}$, one has to end up with Newton Euler equations. Pre-multiplying Eq. (29) with ${\mathbf {L}}$ yields

31 $$\begin{aligned} 4{\mathbf {J}}{\mathbf {L}}\ddot{\mathbf{p}}+8{\mathbf {L}}{\dot{\mathbf{L}}}{}^{T}{\mathbf {J}}{\mathbf {L}}\dot{\mathbf{p}} = 2\overline{\mathbf {n}} \end{aligned}$$

where advantage has been used of the fact that ${\mathbf {L}}{\mathbf {p}}=\mathbf {0}$ and ${\mathbf {L}}{\mathbf {L}}^{T}=\mathbf {I}$. By using the definitions of Eqs. (16, 17), one can transform Eq. (31) to the well-known Newton-Euler equations. The main difference between the equations of motion of Eq. (29) and those of Eq. (23) is given by the fact that some Euler parameter identities have been used for simplifying d’Alembert’s principle. This simplification, which in fact could be interpreted as a modification of the gyroscopic moment, has been demonstrated above by transforming Eq. (27) into Eq. (28).

One might expect that another form of the equations of motion is obtained after setting $\lambda =0$ in Eq. (23), yielding

32 $$\begin{aligned} 4{\mathbf {L}}^{T}{\mathbf {J}}{\mathbf {L}}\ddot{\mathbf{p}}+8{\mathbf {L}}^{T}{\mathbf {L}}{\dot{\mathbf{L}}}{}^{T}{\mathbf {J}}{\mathbf {L}}\dot{\mathbf{p}} = 2{\mathbf {L}}^{T}\overline{\mathbf {n}} \end{aligned}$$

Since we still have four equations for ${\mathbf {p}}$ but no additional variable $\lambda$, it seems that the constraint equation is not needed anymore. However, this is not the case, because Eq. (32) also results from multiplying Eq. (25) with the $4\times 3$ matrix ${\mathbf {L}}^T$. Thus, by linear combination, we have just produced four from three equations, which, of course, are linear dependent. Nevertheless, knowing that $\lambda =0$ in Eq. (23) might be helpful to control the quality of a numerical solution of Eq. (23).

Summing up, we found three different forms of the equations of motion for Euler parameters, given by Eqs. (23), (25) and (29). All of them must be solved in combination with the constraint equation ${\mathbf {p}}^T {\mathbf {p}} = 1$. It seems that the most complicated form is Eq. (23) since it contains fifth-order terms in the Euler parameters and a Lagrange multiplier. In Eq. (25), the multiplier has been eliminated and the terms are of fourth order in the Euler parameters. Finally, Eq. (29) contains only third-order terms in the Euler parameters and a Lagrange multiplier.

There exist different strategies for solving the constrained rotational equations of motion when using Euler parameters. One approach is based on differentiating the kinematic Euler parameter constraint twice with respect to time and on using the augmented form of the equations of motion. This solution procedure is also known as index reduction and will be explained in brief in the remainder of this section. Another solution strategy is based on the elimination of the Lagrange multiplier and is explained in detail in [17]. If the kinematic Euler parameter constraint, see Eq. (4), is differentiated twice with respect to time, we obtain

33 $$\begin{aligned} {\mathbf {p}}^{T}\ddot{\mathbf{p}}+\dot{\mathbf{p}}^{T}\dot{\mathbf{p}}=0 \end{aligned}$$

With Eq. (23), we end up with the following set of equations for ${\mathbf {p}}$ and $\lambda$:

34 $$\begin{aligned} \left[ \begin{array}{c@{\quad }c} 4{\mathbf {L}}^{T}{\mathbf {J}}{\mathbf {L}} &{} {\mathbf {p}} \\ {\mathbf {p}}^{T} &{} 0 \end{array}\right] \left[ \begin{array}{c} \ddot{\mathbf{p}} \\ \lambda \end{array}\right] = \left[ \begin{array}{c} -8{\mathbf {L}}^{T}{\mathbf {L}}{\dot{\mathbf{L}}}{}^{T}{\mathbf {J}}{\mathbf {L}}\dot{\mathbf{p}} + 2{\mathbf {L}}^{T}\overline{\mathbf {n}}\\ -\dot{\mathbf{p}}^{T}\dot{\mathbf{p}} \end{array}\right] \end{aligned}$$

The inverse of the augmented mass matrix on the left side reads [6]

35 $$\begin{aligned} \left[ \begin{array}{c@{\quad }c} 4{\mathbf {L}}^{T}{\mathbf {J}}{\mathbf {L}} &{}{\mathbf {p}} \\ {\mathbf {p}}^{T} &{} 0 \end{array}\right] ^{-1} = \left[ \begin{array}{c@{\quad }c} \textstyle \frac{1}{4}{\mathbf {L}}^{T}{\mathbf {J}}^{-1}{\mathbf {L}} &{}{\mathbf {p}} \\ {\mathbf {p}}^{T} &{} 0 \end{array}\right] \end{aligned}$$

Finally, Eqs. (34) and (35) yield

36 $$\begin{aligned} \left[ \begin{array}{c} \ddot{\mathbf{p}} \\ \lambda \end{array}\right] = \left[ \begin{array}{c@{\quad }c} \textstyle \frac{1}{4}{\mathbf {L}}^{T}{\mathbf {J}}^{-1}{\mathbf {L}} &{} {\mathbf {p}} \\ {\mathbf {p}}^{T} &{} 0 \end{array}\right] \left[ \begin{array}{c} -8{\mathbf {L}}^{T}{\mathbf {L}}{\dot{\mathbf{L}}}{}^{T}{\mathbf {J}}{\mathbf {L}}\dot{\mathbf{p}}+ 2{\mathbf {L}}^{T}\overline{\mathbf {n}}\\ -\dot{\mathbf{p}}^{T}\dot{\mathbf{p}} \end{array}\right] \end{aligned}$$

If we use the relation ${\mathbf {L}}{\mathbf {p}}=\mathbf {0}$, we notice from the second line of Eq. (36) that

37 $$\begin{aligned} \lambda = 0 \end{aligned}$$

showing once again that the Lagrange multiplier $\lambda$ in Eq. (23) is zero. Moreover, the first line of Eq. (36) is a set of four non-redundant equations determining the Euler parameters:

38 $$\begin{aligned} \ddot{\mathbf{p}} = -2{\mathbf {L}}^{T}{\mathbf {J}}^{-1}{\mathbf {L}}{\dot{\mathbf{L}}}{}^{T}{\mathbf {J}}{\mathbf {L}}\dot{\mathbf{p}} +\frac{1}{2}{\mathbf {L}}^{T}{\mathbf {J}}^{-1}\overline{\mathbf {n}} -{\mathbf {p}} \dot{\mathbf{p}}^{T}\dot{\mathbf{p}} \end{aligned}$$

where advantage has been used of ${\mathbf {L}}{\mathbf {L}}^T=\mathbf {I}$.

In order to complete this section, in a last step, we solve the augmented form of the equations of motion of Eq. (29), i.e.,

39 $$\begin{aligned} \left[ \begin{array}{c@{\quad }c} 4{\mathbf {L}}^{T}{\mathbf {J}}{\mathbf {L}} &{} {\mathbf {p}} \\ {\mathbf {p}}^{T} &{} 0 \end{array}\right] \left[ \begin{array}{c} \ddot{\mathbf{p}} \\ \lambda \end{array}\right] = \left[ \begin{array}{c} -8{\dot{\mathbf{L}}}{}^{T}{\mathbf {J}}{\mathbf {L}}\dot{\mathbf{p}} + 2{\mathbf {L}}^{T}\overline{\mathbf {n}}\\ -\dot{\mathbf{p}}^{T}\dot{\mathbf{p}} \end{array}\right] \end{aligned}$$

Using the inverse of the augmented mass matrix of Eq. (35), we obtain

40 $$\begin{aligned} \left[ \begin{array}{c} \ddot{\mathbf{p}} \\ \lambda \end{array}\right] = \left[ \begin{array}{c} \textstyle -2{\mathbf {L}}^{T}{\mathbf {J}}^{-1}{\mathbf {L}}{\dot{\mathbf{L}}}^{T}{\mathbf {JL}}\dot{\mathbf{p}}+ \frac{1}{2}{\mathbf {L}}^{T}{\mathbf {J}}^{-1}\overline{\mathbf {n}}-{\mathbf {p}}\dot{\mathbf{p}}^{T}\dot{\mathbf{p}} \\ -8{\mathbf {p}}^{T}{\dot{\mathbf{L}}}^{T}{\mathbf {JL}}\dot{\mathbf{p}} \end{array}\right] \end{aligned}$$

where the fact has been used that ${\mathbf {L}}{\mathbf {L}}^T=\mathbf {I}$ and that ${\mathbf {L}}{\mathbf {p}}=\mathbf {0}$. The Euler parameter accelerations of Eq. (40) are the same as in Eq. (38). One can also find the formula for the Euler parameter accelerations in [17]. However, the value of the Lagrange multiplier is not zero when using the equations of motion of Eq. (29), see Eq. (30) for the nonzero value of the Lagrange multiplier.

## A detailed discussion on the generalized external force vector

In the last section, different forms of the rotational equations of motion of a rigid body have been presented by manipulating the gyroscopic moment. There, less attention has been paid to the generalized external force vector. The present section focuses on this issue in detail. It will be demonstrated that another form of the equations of motion is obtained by keeping the gyroscopic moment in its original form given by the second term on the left-hand side of Eq. (23), and by manipulating the generalized external force vector. This also will end up in a Lagrange multiplier value different from zero, see Sect. 4.3 for this purpose.

In the last section, we assumed that no pure moments $\overline{\mathbf {m}}$ act on the body and this assumption will hold on in the present section. Thus, the only moment that acts on the rigid body results from an externally applied force vector ${\mathbf {f}}$, i.e.,

41 $$\begin{aligned} \overline{\mathbf {n}}=\widetilde{\overline{\mathbf {u}}}^{p}\overline{{\mathbf {f}}}=\widetilde{\overline{\mathbf {u}}}^{p}\mathbf {A}^{T}{\mathbf {f}} \end{aligned}$$

where the position of application point of the force vector ${\mathbf {f}}$ is given by

42 $$\begin{aligned} \mathbf {r}^{p}=\mathbf {R}+\mathbf {A}\overline{\mathbf {u}}^{p} \end{aligned}$$

The generalized rotational force vector is represented by the right-hand side of Eq. (23), i.e.,

43 $$\begin{aligned} \mathbf {Q}_{p} = 2{\mathbf {L}}^{T}\overline{\mathbf {n}} \end{aligned}$$

Substituting Eq. (41) into Eq. (43) yields

44 $$\begin{aligned} \mathbf {Q}_{p} = 2{\mathbf {L}}^{T}\widetilde{\overline{\mathbf {u}}}^{p}\mathbf {A}^{T}{\mathbf {f}} \end{aligned}$$

The latter form of the generalized torque vector can also be found in [5]. In the following subsection, an alternative approach for computing the generalized external force vector will be presented.

### Derivation of the generalized force vector based on the principle of virtual work

One can also obtain the generalized rotational force vector resulting from the physical force vector ${\mathbf {f}}$ by using the principle of virtual work. The virtual work of an externally applied force ${\mathbf {f}}$ is defined as

45 $$\begin{aligned} \delta W_{e}={\mathbf {f}}^{T}\delta \mathbf {r}^{p} \end{aligned}$$

which can be written in terms of the generalized coordinates $\mathbf {R}$ and ${\mathbf {p}}$ as

46 $$\begin{aligned} \delta W_{e}=\left[ \begin{array}{cc} \left( \mathbf {Q}_{R}\right) ^{T}&\quad \left( \mathbf {Q}_{p}\right) ^{T}\end{array}\right] \left[ \begin{array}{c} \delta \mathbf {R}\\ \delta {\mathbf {p}} \end{array}\right] \end{aligned}$$

where $\mathbf {Q}_{R}$ represents the generalized force vector associated with the translational coordinates and $\mathbf {Q}_{p}$ is the generalized force vector associated with the generalized rotational coordinates.

For the evaluation of vectors $\mathbf {Q}_{R}$ and $\mathbf {Q}_{p}$, the total differential of Eq. (42) has to be computed. Due to the fact that the body under consideration is a rigid body and, therefore, ${\dot{\overline{\mathbf{u}}}}^p=\mathbf {0}$, the first time derivative of Eq. (42) is given by

47 $$\begin{aligned} \dot{\mathbf{r}}^{p}=\dot{\mathbf{R}}+\dot{\mathbf{A}}\overline{\mathbf {u}}^{p} \end{aligned}$$

Using the fact that $\dot{\mathbf{A}}=\mathbf {A}{\widetilde{\overline{\varvec{\omega }}}}$, which follows from Eq. (15), one can write the above equation also as

48 $$\begin{aligned} \dot{\mathbf{r}}^{p}=\dot{\mathbf{R}}-\mathbf {A}\widetilde{\overline{\mathbf {u}}}^{p}{\overline{\varvec{\omega }}} \end{aligned}$$

where the fact has been used that for two arbitrary vectors $\mathbf {y}$ and $\mathbf {z}$ the following relation holds

49 $$\begin{aligned} \widetilde{\mathbf {y}}\mathbf {z}=-\widetilde{\mathbf {z}}\mathbf {y} \end{aligned}$$

Substituting Eq. (17) into Eq. (48) leads to

50 $$\begin{aligned} \dot{\mathbf{r}}^{p}=\dot{\mathbf{R}}+\mathbf {B}_{1}\dot{\mathbf{p}} \end{aligned}$$

where the $3\times 4$ matrix $\mathbf {B}_{1}$ is defined as

51 $$\begin{aligned} \mathbf {B}_{1}=- 2\mathbf {A}\widetilde{\overline{\mathbf {u}}}^{p}{\mathbf {L}} \end{aligned}$$

In a similar way as $\dot{\mathbf {r}}^{p}$, we can express the virtual displacement $\delta \mathbf {r}^{p}$ as

52 $$\begin{aligned} \delta \mathbf {r}^{p}=\delta \mathbf {R}+\mathbf {B}_{1}\delta {\mathbf {p}} \end{aligned}$$

An alternative form of the total differential of Eq. (42) can be obtained by using

53 $$\begin{aligned} \dot{\mathbf{A}}\overline{\mathbf {u}}^{p}=\frac{\partial \mathbf {A}\overline{\mathbf {u}}^{p}}{\partial {\mathbf {p}}}\dot{\mathbf{p}} \end{aligned}$$

We may define the $3\times 4$ matrix $\mathbf {B}_{2}$ to be

54 $$\begin{aligned} \mathbf {B}_{2}=\frac{\partial \mathbf {A}\overline{\mathbf {u}}^{p}}{\partial {\mathbf {p}}} \end{aligned}$$

With the use of this equation and Eq. (53), (47) leads to

55 $$\begin{aligned} \dot{\mathbf{r}}^{p}=\dot{\mathbf{R}}+\mathbf {B}_{2}\dot{\mathbf{p}} \end{aligned}$$

or in terms of virtual displacements

56 $$\begin{aligned} \delta \mathbf {r}^{p}=\delta \mathbf {R}+\mathbf {B}_{2}\delta {\mathbf {p}} \end{aligned}$$

A direct comparison of the matrices $\mathbf {B}_{1}$ and $\mathbf {B}_{2}$ shows that

57 $$\begin{aligned} \mathbf {B}_{1}\ne \mathbf {B}_{2} \end{aligned}$$

At this point, it should be noted that the matrices $\mathbf {B}_{1}$ and $\mathbf {B}_{2}$ are identical if three-dimensional parametrization groups, e.g., Euler angles, are used as rotational coordinates. However, when using Euler parameters, the matrices are not identical. Without any further knowledge on the latter two matrices, we obtain two different forms of the rotational generalized force vector when using the principle of virtual work. The first form is obtained by substituting Eq. (52) into Eq. (45) and by considering only the rotational part (i.e., we set $\delta \mathbf {R}=0$) which leads to

58 $$\begin{aligned} \mathbf {Q}_{p,1}=\mathbf {B}_{1}^{T}{\mathbf {f}} \end{aligned}$$

Since $\mathbf {B}_{1}=- 2\mathbf {A}\widetilde{\overline{\mathbf {u}}}^{p}{\mathbf {L}}$, one can show that

59 $$\begin{aligned} \mathbf {Q}_{p,1} =2{\mathbf {L}}^T\widetilde{\overline{\mathbf {u}}}^p\mathbf {A}^T{\mathbf {f}} \end{aligned}$$

which can be written under the usage of Eqs. (43) and (44) also as

60 $$\begin{aligned} \mathbf {Q}_{p,1} = \mathbf {Q}_p = 2{\mathbf {L}}^T\overline{\mathbf {n}} \end{aligned}$$

Alternatively, when using Eq. (56), we may write the rotational part of the virtual work of the externally applied force as

61 $$\begin{aligned} \delta W_{e}= {\mathbf {f}}^{T}\mathbf {B}_{2}\delta {\mathbf {p}}= \mathbf {Q}_{p,2}^{T}\delta {\mathbf {p}} \end{aligned}$$

such that

62 $$\begin{aligned} \mathbf {Q}_{p,2}=\mathbf {B}_{2}^{T}{\mathbf {f}} \end{aligned}$$

Thus, we end up with two forms for the generalized external force vector associated with the generalized rotational coordinates. The first form is given by Eq. (44) and is exactly the same vector as the vector $\mathbf {Q}_{p,1}$ defined in Eq. (58). The second form is given by the vector $\mathbf {Q}_{p,2}$ of Eq. (62). A direct comparison shows that

63 $$\begin{aligned} \mathbf {Q}_{p,1}\ne \mathbf {Q}_{p,2} \end{aligned}$$

The two different forms of the generalized torque vectors have been implemented for an arbitrarily chosen force vector, and they lead to the same orientation of the body in space. However, the value of the Lagrange multipliers is different for the two formulations. This issue will be discussed in detail in the next subsection.

### Some investigations on the matrices $\mathbf {B}_{1}$ and $\mathbf {B}_{2}$

In order to be able to show that both generalized torque vectors $\mathbf {Q}_{p,1}$ and $\mathbf {Q}_{p,2}$ lead to the same orientation of the body but to different values of the Lagrange multiplier, the matrices $\mathbf {B}_{1}$ and $\mathbf {B}_{2}$ are carefully examined in the present section. Let us start with the matrix $\mathbf {B}_{2}$.

The matrix $\mathbf {B}_{2}$ is defined as the partial derivative of the matrix product $\mathbf {A}\overline{\mathbf {u}}^{p}$ with respect to the Euler parameter vector ${\mathbf {p}}$, see Eq. (54), and can be expanded by using the definition of the rotation matrix of Eq. (5) as [14]:

64 $$\begin{aligned} \mathbf {B}_{2}= & {} \frac{\partial \mathbf {A}\overline{\mathbf {u}}^{p}}{\partial {\mathbf {p}}} =\frac{\partial }{\partial {\mathbf {p}}}\left[ \left( 2e_{0}^{2} -1\right) \overline{\mathbf {u}}^{p} +2\mathbf {e}\mathbf {e}^{T}\overline{\mathbf {u}}^{p} +2e_{0}{\widetilde{\mathbf{e}}}\,\overline{\mathbf {u}}^{p}\right] \nonumber \\= & {} 2\left[ 2e_{0}\overline{\mathbf {u}}^{p}+{\widetilde{\mathbf{e}}}\,\overline{\mathbf {u}}^{p} , \quad \mathbf {e}^{T}\overline{\mathbf {u}}^{p}\mathbf {I}+\mathbf {e}\left( \overline{\mathbf {u}}^{p}\right) ^{T}-e_{0}\widetilde{\overline{\mathbf {u}}}^{p}\right] \end{aligned}$$

Since for any two vectors $\mathbf {y}$ and $\mathbf {z}$ one can verify by direct calculation that

65 $$\begin{aligned} \widetilde{\mathbf {y}}\,\widetilde{\mathbf {z}}=\mathbf {z}\mathbf {y}^{T}-\mathbf {y}^{T}\mathbf {z}\mathbf {I} \end{aligned}$$

we can write Eq. (64) also as

66 $$\begin{aligned} \mathbf {B}_{2}= & {} \frac{\partial \mathbf {A}\overline{\mathbf {u}}^{p}}{\partial {\mathbf {p}}} \nonumber \\= & {} 2\left[ 2e_{0}\overline{\mathbf {u}}^{p}+{\widetilde{\mathbf{e}}}\,\overline{\mathbf {u}}^{p}, \quad \overline{\mathbf {u}}^{p}\mathbf {e}^{T}-{\widetilde{\mathbf{e}}}\,\widetilde{\overline{\mathbf {u}}}^{p}+\mathbf {e}\left( \overline{\mathbf {u}}^{p}\right) ^{T} -e_{0}\widetilde{\overline{\mathbf {u}}}^{p}\right] \nonumber \\= & {} 2\left[ -\mathbf {e}, \quad \widetilde{\mathbf{e}}+e_{0}\mathbf {I}\right] \left[ \begin{array}{l@{\quad }l} 0 &{} -\left( \overline{\mathbf {u}}^{p}\right) ^{T}\\ \overline{\mathbf {u}}^{p} &{} -\widetilde{\overline{\mathbf {u}}}^{p} \end{array}\right] \nonumber \\&\quad +\,2\left[ e_{0}\overline{\mathbf {u}}^{p}, \quad \overline{\mathbf {u}}^{p}\mathbf {e}^{T}\right] \end{aligned}$$

We may define the $4\times 4$ skew-symmetric matrix ${\mathbf {H}}$ to be

67 $$\begin{aligned} {\mathbf {H}}=\left[ \begin{array}{l@{\quad }l} 0 &{} -\left( \overline{\mathbf {u}}^{p}\right) ^{T}\\ \overline{\mathbf {u}}^{p} &{} -\widetilde{\overline{\mathbf {u}}}^{p} \end{array}\right] \end{aligned}$$

With the use of this definition and Eqs. (2, 8), (66) can be expressed in compact form as

68 $$\begin{aligned} \mathbf {B}_{2}=2{\mathbf {G}}{\mathbf {H}}+2\overline{\mathbf {u}}^{p}{\mathbf {p}}^{T} \end{aligned}$$

Thus, the generalized external force vector associated with the rotational coordinates of Eq. (62) can be written as

69 $$\begin{aligned} \mathbf {Q}_{p,2}=2{\mathbf {H}}^{T}{\mathbf {G}}^{T}{\mathbf {f}}+2{\mathbf {p}}\left( \overline{\mathbf {u}}^{p}\right) ^{T}{\mathbf {f}} \end{aligned}$$

By taking the first time derivative of Eq. (4), one can verify that the second part of the right-hand side of the previous equation is orthogonal to $\dot{\mathbf{p}}$. It can be noted that this part does not contribute to the orientation of the body but has an influence on the Lagrange multiplier if implemented. The fact that its implementation has no influence on the orientation of the body can be demonstrated by substituting the expression of $\mathbf {Q}_{p,2}$ into Eq. (61), i.e.,

70 $$\begin{aligned} \delta W_{e}= \mathbf {Q}_{p,2}^{T}\delta {\mathbf {p}}= 2{\mathbf {f}}^{T}{\mathbf {G}}{\mathbf {H}}\delta {\mathbf {p}}+2{\mathbf {f}}^{T}\overline{\mathbf {u}}^{p}{\mathbf {p}}^{T}\delta {\mathbf {p}} \end{aligned}$$

The last term on the right-hand side of the previous equation vanishes due to the orthogonality of ${\mathbf {p}}$ and $\delta {\mathbf {p}}$. Thus, the second part of the right-hand side of Eq. (69) does not play any role in the rotational dynamics of the body.

Now, we try to split the matrix $\mathbf {B}_{1}$ of Eq. (51) into two components in order to show that the generalized force vector associated with the rotational coordinates given by Eq. (58) consists of a part that is orthogonal to $\dot{\mathbf{p}}$ and therefore does not influence the rotational dynamics, and a second part that causes a rotation of the body.

The product ${\mathbf {L}}{\mathbf {H}}$ may be evaluated as follows:

71 $$\begin{aligned} {\mathbf {L}}{\mathbf {H}}= & {} \Big [-\mathbf {e},\quad -{\widetilde{\mathbf{e}}}+e_{0}\mathbf {I}\Big ] \left[ \begin{array}{l@{\quad }l} 0 &{} -\left( \overline{\mathbf {u}}^{p}\right) ^{T}\\ \overline{\mathbf {u}}^{p} &{}-\widetilde{\overline{\mathbf {u}}}^{p} \end{array}\right] \nonumber \\= & {} \left[ -{\widetilde{\mathbf{e}}}\,\overline{\mathbf {u}}^{p}+e_{0}\overline{\mathbf {u}}^{p}, \quad \mathbf {e}\left( \overline{\mathbf {u}}^{p}\right) ^{T}+\widetilde{\mathbf{e}}\,\widetilde{\overline{\mathbf {u}}}^{p}-e_{0}\widetilde{\overline{\mathbf {u}}}^{p}\right] \nonumber \\= & {} -\widetilde{\overline{\mathbf {u}}}^{p}\Big [-\mathbf {e},\quad -{\widetilde{\mathbf{e}}}+e_{0}\mathbf {I}\Big ]+\left[ e_{0}\overline{\mathbf {u}}^{p},\quad \overline{\mathbf {u}}^{p}\mathbf {e}^{T}\right] \end{aligned}$$

or in a more compact form as

72 $$\begin{aligned} {\mathbf {L}}{\mathbf {H}}=-\widetilde{\overline{\mathbf {u}}}^{p}{\mathbf {L}}+\overline{\mathbf {u}}^{p}{\mathbf {p}}^{T} \end{aligned}$$

Using Eqs. (7, 72), the matrix $\mathbf {B}_{1}$ of Eq. (51) can be rewritten as

73 $$\begin{aligned} \mathbf {B}_{1}=2{\mathbf {G}}{\mathbf {L}}^{T}{\mathbf {L}}{\mathbf {H}}-2{\mathbf {G}}{\mathbf {L}}^{T}\overline{\mathbf {u}}^{p}{\mathbf {p}}^{T} \end{aligned}$$

Substituting Eq. (11) into Eq. (73) yields

74 $$\begin{aligned} \mathbf {B}_{1}=2{\mathbf {G}}{\mathbf {H}}-2{\mathbf {G}}{\mathbf {L}}^{T}\overline{\mathbf {u}}^{p}{\mathbf {p}}^{T} \end{aligned}$$

where advantage has been taken of the fact that ${\mathbf {G}}{\mathbf {p}}=\mathbf {0}$, see Eq. (12). Thus, we can also write the rotational generalized external force vector of Eq. (58) as

75 $$\begin{aligned} \mathbf {Q}_{p,1}=2{\mathbf {H}}^{T}{\mathbf {G}}^{T}{\mathbf {f}}-2{\mathbf {p}}\left( \overline{\mathbf {u}}^{p}\right) ^{T}{\mathbf {L}}{\mathbf {G}}^{T}{\mathbf {f}} \end{aligned}$$

The latter equation informs us that—as it is the case for $\mathbf {Q}_{p,2}$—the generalized torque vector $\mathbf {Q}_{p,1}$ consists of a component in the direction of ${\mathbf {p}}$ and a second component which plays a role in the rotational dynamics of a rigid body. The form of $\mathbf {Q}_{p,2}$ is much simpler than the form of $\mathbf {Q}_{p,1}$ since it just depends linearly on the Euler parameters and, thus, yields to a Jacobian which does not depend on the Euler parameters. In contrast, $\mathbf {Q}_{p,1}$ depends cubically on the Euler parameters.

### Incorporation of the generalized torque into the rotational equations of motion

Although it has been demonstrated in Sect. 3 that one can simplify Eq. (20) by using the fact that ${\mathbf {L}}^{T}{\mathbf {L}}=\mathbf {I}_{4}-{\mathbf {p}}{\mathbf {p}}^{T}$ resulting in a simpler form of d’Alembert’s principle in Eq. (28), the starting point of our investigation is d’Alembert’s principle in the form of Eq. (20), i.e.,

76 $$\begin{aligned} \delta {\mathbf {p}}^T\left( 4{\mathbf {L}}^{T}{\mathbf {J}}{\mathbf {L}}\ddot{\mathbf{p}}+8{\mathbf {L}}^{T}{\mathbf {L}}{\dot{\mathbf{L}}}{}^{T}{\mathbf {J}}{\mathbf {L}}\dot{\mathbf{p}}-2{\mathbf {L}}^{T}\overline{\mathbf {n}}\right) = 0 \end{aligned}$$

The reason for doing so is that Eq. (20) leads to the most common formulation of the rotational equations of motion where the Lagrange multiplier is zero and the gyroscopic moment is given in its original form. The focus in the present section lies on the incorporation of the external generalized moment vector into the equations of motion and its effect on the value of the Lagrange multiplier.

Using the definition of $\overline{\mathbf {n}}$ of Eq. (41), we get for Eq. (20)

77 $$\begin{aligned} \delta {\mathbf {p}}^T\left( 4{\mathbf {L}}^{T}{\mathbf {J}}{\mathbf {L}}\ddot{\mathbf{p}}+8{\mathbf {L}}^{T}{\mathbf {L}}{\dot{\mathbf{L}}}^{T}{\mathbf {J}}{\mathbf {L}}\dot{\mathbf{p}}-2{\mathbf {L}}^{T}\widetilde{\overline{\mathbf {u}}}^{p}\mathbf {A}^{T}{\mathbf {f}}\right) = 0 \end{aligned}$$

whereby the variation $\delta {\mathbf {p}}$ is constrained by the relation $\delta {\mathbf {p}}^T {\mathbf {p}} = 0$. Equation (77) can be rewritten by using Eqs. (58) and (59) as

78 $$\begin{aligned} \delta {\mathbf {p}}^T\left( 4{\mathbf {L}}^{T}{\mathbf {J}}{\mathbf {L}}\ddot{\mathbf{p}}+8{\mathbf {L}}^{T}{\mathbf {L}}{\dot{\mathbf{L}}}{}^{T}{\mathbf {J}}{\mathbf {L}}\dot{\mathbf{p}}-\mathbf {B}_1^{T}{\mathbf {f}}\right) = 0 \end{aligned}$$

Without any further knowledge on the matrix $\mathbf {B}_1$ and by adding the zero term $\delta {\mathbf {p}}^T {\mathbf {p}}\lambda$ to Eq. (78), we end up with the following equations of motion

79 $$\begin{aligned} 4{\mathbf {L}}^{T}{\mathbf {J}}{\mathbf {L}}\ddot{\mathbf{p}}+8{\mathbf {L}}^{T}{\mathbf {L}}{\dot{\mathbf{L}}}{}^{T}{\mathbf {J}}{\mathbf {L}}\dot{\mathbf{p}} + {\mathbf {p}}\lambda = \mathbf {B}_1^{T}{\mathbf {f}} \end{aligned}$$

Clearly, the latter equation has to be constrained by the kinematic Euler parameter constraint. Since $\mathbf {B}_1^{T}{\mathbf {f}} = 2{\mathbf {L}}^{T}\mathbf {n}$, see Eqs. (58) and (60) for this relation, the latter equation is equal to Eq. (23). Thus, for this case the Lagrange multiplier is zero and the Euler parameter accelerations are given by Eqs. (38). However, as demonstrated in Sect. 4.2, we know that the generalized force vector $\mathbf {Q}_{p1}= \mathbf {B}_1^{T}{\mathbf {f}}$ can be split into two parts, see Eq. (75). Let us therefore include this information in the d’Alembert’s principle of Eqs. (78) yielding:

80 $$\begin{aligned}&\delta {\mathbf {p}}^T\left( 4{\mathbf {L}}^{T}{\mathbf {J}}{\mathbf {L}}\ddot{\mathbf{p}}+8{\mathbf {L}}^{T}{\mathbf {L}}{\dot{\mathbf{L}}}{}^{T}{\mathbf {J}}{\mathbf {L}}\dot{\mathbf{p}}-2{\mathbf {H}}^{T}{\mathbf {G}}^{T}{\mathbf {f}} \right. \nonumber \\&\quad \left. +\,2{\mathbf {p}}\left( \overline{\mathbf {u}}^{p}\right) ^{T}{\mathbf {L}}{\mathbf {G}}^{T}{\mathbf {f}}\right) = 0 \end{aligned}$$

Since $\delta {\mathbf {p}}^T {\mathbf {p}} = 0$, the latter equation can be rewritten in a simpler form as

81 $$\begin{aligned} \delta {\mathbf {p}}^T\left( 4{\mathbf {L}}^{T}{\mathbf {J}}{\mathbf {L}}\ddot{\mathbf{p}}+8{\mathbf {L}}^{T}{\mathbf {L}}{\dot{\mathbf{L}}}{}^{T}{\mathbf {J}}{\mathbf {L}}\dot{\mathbf{p}}-2{\mathbf {H}}^{T}{\mathbf {G}}^{T}{\mathbf {f}}\right) = 0 \end{aligned}$$

As it has been explained in Sect. 3, it is not possible to equate the expression in brackets to zero, because the Euler parameters are not independent quantities. Therefore, we first have to add the zero term $\delta {\mathbf {p}}^T {\mathbf {p}}\lambda$ to Eq. (81), which finally results in

82 $$\begin{aligned} 4{\mathbf {L}}^{T}{\mathbf {J}}{\mathbf {L}}\ddot{\mathbf{p}}+8{\mathbf {L}}^{T}{\mathbf {L}}{\dot{\mathbf{L}}}{}^{T}{\mathbf {J}}{\mathbf {L}}\dot{\mathbf{p}} + {\mathbf {p}}\lambda = 2{\mathbf {H}}^{T}{\mathbf {G}}^{T}{\mathbf {f}} \end{aligned}$$

By differentiating the kinematic Euler parameter constraint twice with respect to time, the augmented form of the equations of motion can be written as

83 $$\begin{aligned}&\left[ \begin{array}{c@{\quad }c} 4{\mathbf {L}}^{T}{\mathbf {J}}{\mathbf {L}} &{} {\mathbf {p}} \\ {\mathbf {p}}^{T} &{}0 \end{array}\right] \left[ \begin{array}{c} \ddot{\mathbf{p}} \\ \lambda \end{array}\right] \nonumber \\&\quad = \left[ \begin{array}{c} -8{\mathbf {L}}^{T}{\mathbf {L}}{\dot{\mathbf{L}}}{}^{T}{\mathbf {J}}{\mathbf {L}}\dot{\mathbf{p}}+ 2{\mathbf {H}}^{T}{\mathbf {G}}^{T}{\mathbf {f}}\\ -\dot{\mathbf{p}}^{T}\dot{\mathbf{p}} \end{array}\right] \end{aligned}$$

Using the inverse of the augmented mass matrix of Eq. (35) we finally get for the Lagrange parameter and the Euler parameter accelerations:

84 $$\begin{aligned}&\left[ \begin{array}{c} \ddot{\mathbf{p}} \\ \lambda \end{array}\right] \nonumber \\&\quad = \left[ \begin{array}{c} \textstyle -2{\mathbf {L}}^{T}{\mathbf {J}}^{-1}{\mathbf {L}}{\dot{\mathbf{L}}}^{T}{\mathbf {JL}}\dot{\mathbf{p}}+ \frac{1}{2}{\mathbf {L}}^{T}{\mathbf {J}}^{-1}{\mathbf {L}}{\mathbf {H}}^{T}{\mathbf {G}}^{T}{\mathbf {f}} -{\mathbf {p}}\dot{\mathbf{p}}^{T}\dot{\mathbf{p}} \\ 2{\mathbf {p}}^{T}{\mathbf {H}}^{T}{\mathbf {G}}^{T}{\mathbf {f}} \end{array}\right] \end{aligned}$$

Since $\mathbf {LH}^T \mathbf {G}^T\mathbf {f}=\widetilde{\overline{\mathbf {u}}}^{p}\mathbf {A}^T\mathbf {f}=\overline{\mathbf {n}}$, the Euler parameter accelerations in Eq. (84) are the same as in all other presented forms of the equations of motion, but the Lagrange multiplier takes in this case the value $2{\mathbf {p}}^{T}{\mathbf {H}}^{T}{\mathbf {G}}^{T}{\mathbf {f}}$. This is based on the manipulation of the external force vector.

Following the same procedure as demonstrated above for $\mathbf {Q}_{p,1}$, one gets for the second form of the generalized force vector, i.e., $\mathbf {Q}_{p,2}$, also two different forms of the equations of motion. This is based on the fact that $\mathbf {Q}_{p,2}$ can be separated into two parts, see Eq. (69), whereby the part $2{\mathbf {p}}\left( \overline{\mathbf {u}}^{p}\right) ^{T}{\mathbf {f}}$ can be eliminated already at d’Alembert’s principle level since $\delta {\mathbf {p}}^T {\mathbf {p}}$ is zero.

At this point it should be pointed out once again that all presented forms of the equations of motion must be solved in combination with the constraint equation ${\mathbf {p}}^T {\mathbf {p}} = 1$. Moreover, all forms lead to the same Euler parameter acceleration, thus to the same movement of the body, but the value of the Lagrange multiplier is different. The Lagrange multiplier is only zero, when the most common form, i.e., Eq. (23), is implemented. For this special formulation, the Lagrange multiplier is zero because all generalized moments (inertia, gyroscopic, external) in the equations of motion are orthogonal to the Euler parameter vector ${\mathbf {p}}$, and only the moment resulting from the kinematic Euler parameter constraint, i.e., ${\mathbf {p}}\lambda$, is parallel to the Euler parameter vector. However, by using some Euler parameter identities, it is possible to eliminate some terms at the level of d’Alembert’s principle (manipulating either the gyroscopic moment or the generalized external force vector), and for the resulting equations of motion, the Lagrange multiplier is no longer zero then.

## Conclusion

Various forms of the rotational equations of motion of a rigid body have been presented for the case of using Euler parameters for the rotation. They must be solved in combination with the constraint equation of the Euler parameters. The crucial difference between the various types of equations of motion is given, on the one hand, by their mathematical complexity and, on the other hand, by the value of the Lagrange multiplier. In some cases, the Lagrange multiplier takes the value of zero, which might be helpful to control the quality of the numerical solution. Although it might seem paradoxical at first sight, for the case of using Euler parameters as rotational coordinates, both the gyroscopic moment and the generalized torque vector could be represented in various different forms. They all lead to the same rotation of the body but to different values of the Lagrange multiplier. Although the present paper deals with rigid body dynamics, exactly the same problem occurs if the body under consideration is flexible.
